# Heterophase Engineering Creates a Built‐in Highway to Achieve Local High‐Concentration Phosphorus Doping for Robust K‐Ion Storage

**DOI:** 10.1002/advs.202518687

**Published:** 2025-11-05

**Authors:** Dawei Sha, Yurong You, Yuan Zhang, Long Pan, ZhengMing Sun

**Affiliations:** ^1^ Institute of Technology for Carbon Neutralization College of Electrical Energy and Power Engineering Yangzhou University Yangzhou Jiangsu China; ^2^ School of Materials Science and Engineering Southeast University Nanjing Jiangsu China

**Keywords:** conversion‐type anodes, Heterophase, K‐ion batteries, phosphorus doping, transition metal chalcogenides

## Abstract

Doping is a promising strategy to enhance the K^+^ storage performance of transition metal chalcogenides (TMCs), which are attractive conversion‐type anodes for K‐ion batteries (KIBs) due to their low cost and natural abundance. However, conventional doping approaches are often limited by low doping content or structural degradation at high doping levels, making it highly challenging to achieve efficient doping without compromising structural integrity. Herein, a local high‐concentration doping strategy enabled by heterophase engineering is proposed to overcome this limitation. The boundaries in the heterophase of CoSe_2_/ZnSe act as built‐in highways for dopant diffusion and doping formation, achieving a remarkably high P doping content of 34 at.%. Such local high‐concentration P doping enhances the internal electric field and weakens bonding strength, thereby accelerating K^+^ storage kinetics. Benefiting from these effects, the optimized P‐C/Z@C electrode delivers outstanding electrochemical performance, including 166 mAh g^−1^ at 10.0 A g^−1^ and 180 mAh g^−1^ at 5.0 A g^−1^ after 1400 cycles. Moreover, a full battery paired with a potassium Prussian blue cathode achieves a high energy density of 218 Wh kg^−1^, highlighting its practical feasibility. This work provides a new pathway for enhancing doping efficiency in TMCs and offers valuable insights for designing high‐performance anodes for KIBs.

## Graphical abstract

A local high‐concentration doping enabled by heterophase engineering is proposed for the first time, achieving a remarkable P doping level of 34 at.% and thereby maximizing the advantages of doping for enhancing the K⁺ storage performance of transition metal chalcogenides (TMCs). The local high‐concentration P doping strengthens the electrical field and weakens the bonding strength of TMCs to boost the charge transport kinetics, resulting in the robust K^+^ storage performance.

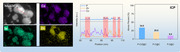

## 1 Introduction

A low‐cost, high‐energy‐density ion storage technique is crucial to meet the increasing demand for large‐scale energy storage systems (LSESSs), which play a critical role in storing intermittent renewable energy and ensuring stable power output.^[^ ^1^ ^]^ Compared with Li‐ion batteries and Na‐ion batteries, K‐ion batteries (KIBs) show distinct advantages for LSESSs due to the abundance of potassium in the earth's crust (Li *vs*. Na *vs*. K: 0.0017 *vs*. 2.3 *vs*. 1.7 wt.%) and its low redox potential (Li^+^/Li *vs*. Na^+^/Na *vs*. K^+^/K: –3.04 *vs*. –2.71 *vs*. –2.93 V). These features endow KIBs with a lower cost than Li‐ion batteries and a higher energy density than Na‐ion batteries.^[^ ^2^, ^3^, ^4^ ^]^ In addition, K^+^ exhibits the weakest Lewis acidity among Li^+^, Na^+^, and K^+^, leading to the smallest Stokes radius and the lowest de‐solvation energy in electrolytes.^[^ ^5^, ^6^, ^7^ ^]^ However, the advancement of KIBs is significantly hindered by the scarcity of anodes that simultaneously deliver high K^+^ specific capacity and long cycling life. In this context, low‐cost transition metal chalcogenides (TMCs: M_a_X_b_, M = V, Co, Zn, In, Bi…; X = S, Se, Te), which possess high theoretical specific capacities owing to their multielectron‐transfer K^+^ storage mechanism, are regarded as promising anode candidates for KIBs. Unfortunately, TMCs face significant challenges, including severe volume change and sluggish storage kinetics upon K^+^ insertion/extraction, resulting in rapid capacity fading and unsatisfactory practical performance.^[^ ^8^ ^]^

Doping has emerged as an effective strategy to enhance the ion storage performance of TMCs by modulating their intrinsic structures and properties—such as charge transportability, ion adsorption capability, and bonding strength—distinct from carbon coating or nanosizing approaches that primarily alter the external environment.^[^ ^9^, ^10^, ^11^ ^]^ Recent studies have further revealed that doping can accelerate ion diffusion in the intercalation stage while facilitating M‐X bond breakage in the subsequent conversion stage, thereby improving the K^+^ storage of TMCs.^[^ ^12^, ^13^ ^]^ However, the unfavorable doping thermodynamics and kinetics have thus far restricted doping levels to relatively low concentrations.^[^ ^14^, ^15^ ^]^ For instance, phosphorus (P) is frequently introduced into TMCs (e.g., sulfides and selenides) to enhance electrical conductivity, ion‐storage capacity, and catalytic activity. Nevertheless, the doping concentration typically remains below 7 at%, resulting in only limited performance gains, as summarized in Figure S1 (Supporting Information).^[^ ^16^, ^17^, ^18^ ^]^ On the other hand, high‐concentration doping atoms may significantly distort and even destroy the structures and morphologies of TMCs, leading to severely detrimental effects on ion storage.^[^ ^15^ ^]^ Therefore, it is imperative to develop novel and effective strategies that enable maximized doping concentration in TMCs while preserving structural and morphological stability, to fully exploit the beneficial effects of doping.

For the first time, we propose a local high‐concentration doping strategy enabled by heterophase engineering to achieve high P‐doping content in TMCs. In this approach, dopant atoms are primarily concentrated at the boundaries of heterophase, where the P content is significantly higher than in regions without boundaries. The doping process involves two key steps: i) diffusion of dopant atoms into the host material, and ii) substitution of host atoms by dopant atoms.^[^ ^15^, ^17^ ^]^ Due to lattice mismatch and electronic orbital reconstruction at the boundaries of heterophase,^[^ ^19^, ^20^, ^21^, ^22^ ^]^ both steps proceed more readily at the boundaries than in bulk regions, leading to local high‐concentration doping. Under these conditions, high doping contents can be achieved without destroying the overall structure and morphology. Such local high‐concentration doping at heterophase amplifies the beneficial effects on K^+^ storage, which cannot be realized by doping or heterophase engineering alone. During the intercalation stage (M_a_X_b_ + *n*K^+^ + *n*e^−^ → K_n_M_a_X_b_), the electric field arising from the work function difference between heterophases is further enhanced by local high‐concentration doping, thereby accelerating K^+^ and electron transport.^[^ ^23^ ^]^ Subsequently, during the conversion stage [K_n_M_a_X_b_ + (2*b* – *n*)K^+^ + (2*b* – *n*)e^−^ → *a*M + *b*K_2_X], the bonding strength at the heterophase is further weakened, facilitating the conversion reaction.^[^ ^13^ ^]^ As a result, the overall K⁺ storage performance, including both rate capability and cycling stability, is significantly improved.

Specifically, in this study, local high‐concentration P doping was achieved by synthesizing CoSe_2_/ZnSe@C (C/Z@C) heterophase with abundant boundaries, enabling a P doping content of 34 at% in the host material (P‐C/Z@C). Theoretical simulations guided that at the CoSe_2_/ZnSe heterophase, the P diffusion energy barrier is lower than that in single phases CoSe_2_@C (C@C) and ZnSe@C (Z@C), facilitating the first step of the doping process (dopant diffusion). Furthermore, the formation energy of P substitution at the boundary is also lower than in C@C or Z@C, promoting the second step (dopant incorporation). As a result, the P at the boundaries of heterophase is higher than that in the bulk phase, suggesting the concentrated P atoms at the boundaries of heterophase. This renders P‐C/Z@C a significantly higher doping content compared to P‐C@C and P‐Z@C. The boundary‐enriched P doping enhances the intrinsic electric field, accelerating charge transport during the intercalation stage. Meanwhile, the bonding strength of Co–Se and Zn–Se near the boundaries is weakened, thereby facilitating the conversion reactions (Co–Se → Co + K_2_Se; Zn–Se → Zn + K_2_Se). These synergistic effects lead to faster K⁺ storage kinetics and a higher K^+^ diffusion coefficient. Importantly, among the doping pairs, the P‐C/Z@C vs. C/Z@C comparison shows the greatest improvement in K^+^ storage performance, surpassing the enhancements observed in P‐C@C vs. C@C and P‐Z@C vs. Z@C. Consequently, P‐C/Z@C delivers outstanding performance, including a rate capacity of 166 mAh g^−1^ at 10.0 A g^−1^ and excellent cycling stability (180 mAh g^−1^ at 5.0 A g^−1^ after 1400 cycles). When coupled with a potassium Prussian blue (KPB) cathode, the full cell achieves a high energy density of 218 Wh kg^−1^, demonstrating its strong potential for practical KIBs.

## 2 Results and Discussion

**Figure 1:**
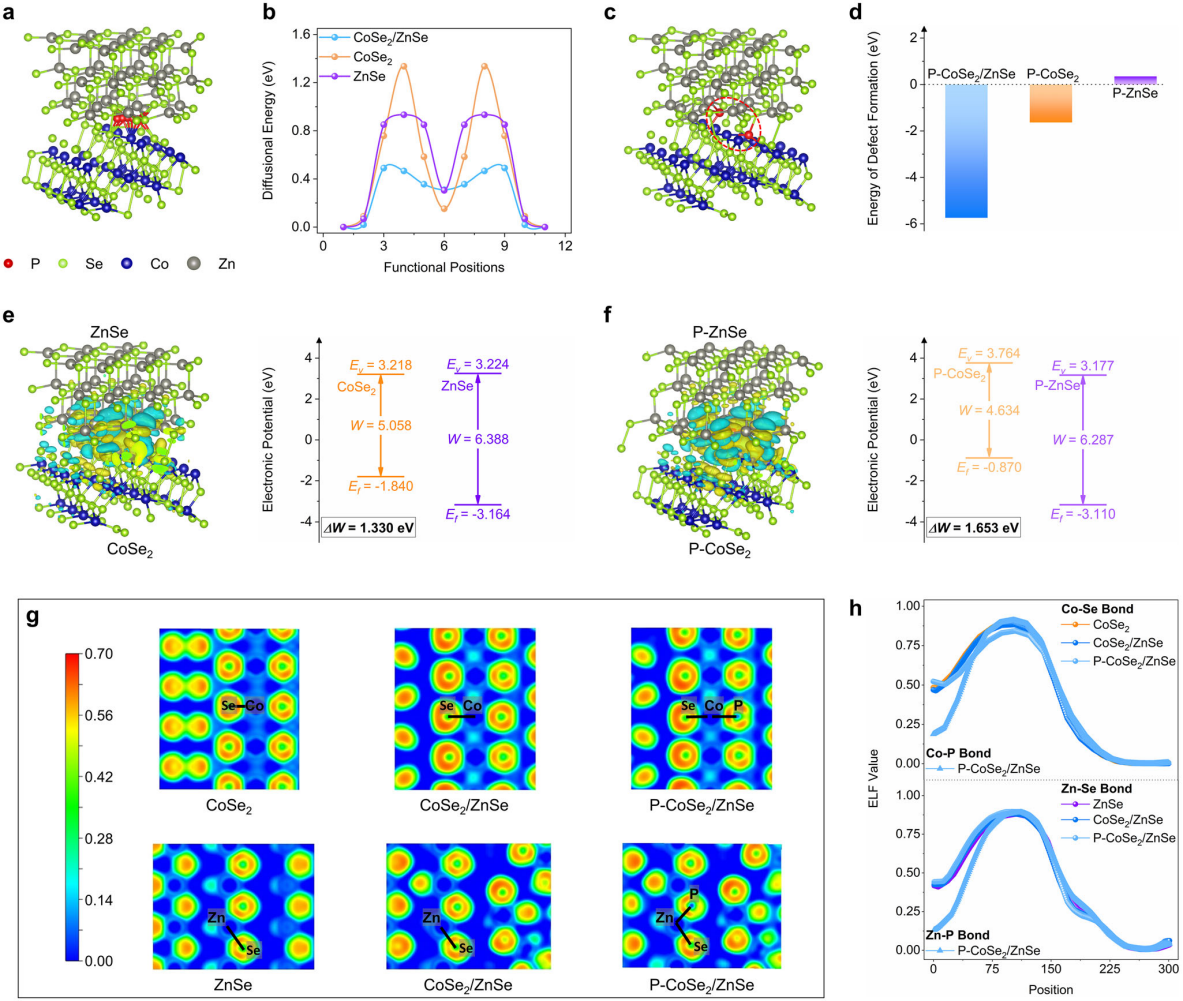
a) P diffusion pathway in CoSe_2_/ZnSe; b) the diffusional energy barriers of P in CoSe_2_, ZnSe, and CoSe_2_/ZnSe; c) atomic structure of P‐CoSe_2_/ZnSe; d) the defect formation energies of P doping; charge density difference and work function of e) CoSe_2_/ZnSe and f) P‐CoSe_2_/ZnSe; g) 3D electron density distribution maps and h) the corresponding ELF values.

Theoretical simulations were initially conducted to investigate the influence of the CoSe_2_/ZnSe heterophase on P doping behavior. Upon doping, P atoms tend to diffuse within the host materials. The optimized diffusion pathways of P in CoSe_2_, ZnSe, and CoSe_2_/ZnSe are shown in Figure S2 (Supporting Information) and **Figure** **1**a. Taking CoSe_2_/ZnSe as an example, the P atom migrates from the most energetically favorable site to a neighboring position. Notably, the CoSe_2_/ZnSe heterophase exhibits the lowest diffusion energy barrier of 0.49 eV, in comparison to 1.34 eV for CoSe_2_ and 0.93 eV for ZnSe (Figure 1b), indicating a facilitated P diffusion process at the CoSe_2_/ZnSe heterophase. Subsequently, P doping is realized by substituting Se atoms in the selenides with P atoms.^[^ ^24^, ^25^ ^]^ The optimized atomic structures of the doped systems (e.g., P‐CoSe_2_, P‐ZnSe, and P‐CoSe_2_/ZnSe) are shown in Figure S3 (Supporting Information) and Figure 1c. These configurations represent the most thermodynamically stable doping sites. The defect formation energies of P doping in the selenides are calculated by the equation E_df_ = E_P‐doped selenides_ – E_selenides_ – E_P_, where E_df_, E_P‐doped selenides_, E_selenides_, and E_P_ correspond to the defect formation energy, the free energy of P‐doped selenides, the free energy of selenides without P doping, and the free energy of P, respectively. The free energies of all samples are shown in Table S1 (Supporting Information). As depicted in Figure 1d, the calculated defect formation energies for P‐ZnSe, P‐CoSe_2_, and P‐CoSe_2_/ZnSe are 0.35, −1.63, and −5.74 eV, respectively, indicating that P doping is most favorable at the CoSe_2_/ZnSe heterophase.^[^ ^26^, ^27^ ^]^ Theoretical simulations reveal that the boundary in the heterophase can act as an efficient doping tunnel, offering a promising strategy to achieve a local high‐concentration doping in the TMCs.

To elucidate the beneficial effects of the local high‐concentration doping on the intercalation and conversion reactions during K^+^ storage, work function analysis and electron localization function (ELF) calculations are conducted. As shown in Figure 1e, the charge density difference and corresponding work functions of the CoSe_2_/ZnSe heterophase reveal significant electronic redistribution at the boundary. In the charge density difference, the yellow and blue regions represent charge depletion and accumulation, respectively. Evidently, electrons transfer from CoSe_2_ to ZnSe, leading to the formation of an intrinsic electric field across the boundary. The work function difference between CoSe_2_ (5.058 eV) and ZnSe (6.388 eV) yields *Δ*W = 1.330 eV, further confirming the presence of this interfacial electric field, which is expected to provide an internal driving force for K^+^ and electron transport during the intercalation process.^[^ ^21^, ^23^ ^]^ Upon P doping, this effect is further amplified. As illustrated in Figure 1f, the charge transfer becomes more pronounced in the P‐CoSe_2_/ZnSe system, accompanied by an increased work function difference (*Δ*W = 1.653 eV), indicating an enhanced intrinsic electric field. This strengthened field is anticipated to facilitate ion/electron transport more effectively during the intercalation stage.

Bonding strength is analyzed through ELF and electron density distribution to understand the impact of local high‐concentration doping on the conversion reaction. Figure 1g presents the 3D electron density maps of the selenides. In these maps, the red regions surrounding Se atoms indicate localized electrons, with deeper red suggesting stronger bonding. No significant change is observed in the Co–Se bond between CoSe_2_ and CoSe_2_/ZnSe. However, after P doping at the phase boundary, the red region around Se atoms in P‐CoSe_2_/ZnSe becomes smaller and lighter, signifying a weakening of the Co–Se bond. Additionally, weak electron localization around P atoms implies the formation of weak Co–P bonds. The corresponding ELF values, shown in Figures 1h and S4 (Supporting Information), quantitatively support these observations. The ELF value of Co–Se and Co–P bonds in P‐CoSe_2_/ZnSe is lower than that of Co–Se bonds in pristine CoSe_2_ and CoSe_2_/ZnSe, indicating reduced bond strength.^[^ ^13^, ^28^, ^29^ ^]^ Similarly, the Zn–P bond also exhibits reduced ELF values after P doping. The ELF value for the Zn‐Se bond is not obviously reduced. These results suggest that P doping introduces low‐bond‐strength sites and weakens the neighboring Co–Se bonds, thus facilitating the conversion reaction by lowering the energy barrier for bond cleavage.

Collectively, these theoretical simulations highlight that the boundary in the heterophase can serve as built‐in doping tunnels to promote P doping (P diffusion and P doping formation). The doped P at the boundary of heterophase can enhance K^+^ storage kinetics to a large degree, where the intrinsic electrical field located at the boundary is enhanced to boost K^+^ intercalation, and the bonding strength of metal‐Se is weakened to facilitate the conversion reaction.

**Figure 2:**
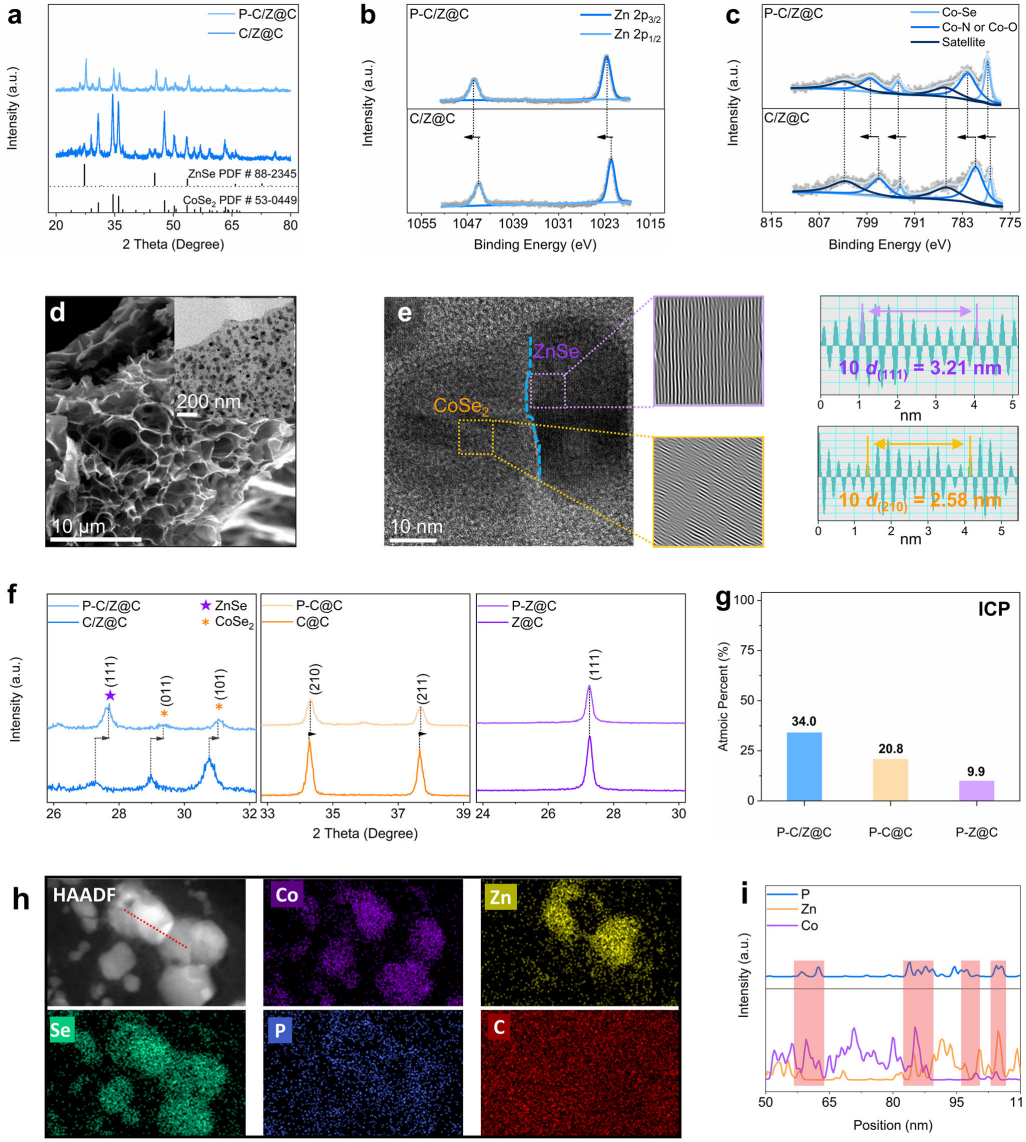
a) XRD patterns of C/Z@C and P‐C/Z@C; high‐resolution XPS spectra of b) Zn 2p and c) Co 2p for C/Z@C and P‐C/Z@C; d) SEM image and inset TEM image of P‐C/Z@C; e) HRTEM image with corresponding IFFT image and lattice spacing; f) comparison of XRD peak shifts; g) P atomic percentage in P‐C/Z@C, P‐C@C, and P‐Z@C; h) HAADF image elemental mappings of P‐C/Z@C; i) elemental distribution profile along the red line in the HAADF image shown in (h).

Encouraged by the positive results from theoretical simulations, a heterophase composite, CoSe_2_/ZnSe@C (denoted as C/Z@C) was designed, where the incorporated carbon enhances the electronic conductivity of the selenides and buffers volume changes during K⁺ insertion and extraction. Subsequently, P was doped into C/Z@C to obtain the P‐doped composite, referred to as P‐C/Z@C. The XRD patterns of C/Z@C and P‐C/Z@C are shown in **Figure** **2**a. No significant phase change is observed after P doping, and both samples exhibit characteristic diffraction peaks corresponding to CoSe_2_ (PDF #53‐0449) and ZnSe (PDF #88‐2345).^[^ ^30^ ^]^ To verify the successful doping of P, high‐resolution XPS analyses are conducted. The full XPS survey scans of P‐C/Z@C and C/Z@C are shown in Figure S5 (Supporting Information). As shown in Figure 2b, the Zn 2p spectrum of P‐C/Z@C displays a positive shift in binding energy (1045.9 eV for Zn 2p_1/2_ and 1022.6 eV for Zn 2p_3/2_) compared to that of C/Z@C (1045.0 and 1021.8 eV, respectively). This shift is attributed to the higher electronegativity of P relative to Se, which leads to reduced electron density around Zn atoms.^[^ ^31^ ^]^ In the high‐resolution Co 2p spectra (Figure 2c), C/Z@C exhibits peaks at 793.4 and 778.4 eV corresponding to Co^2+^ 2p_1/2_ and 2p_3/2_, and at 797.1 and 780.7 eV corresponding to Co^3+^ 2p_1/2_ and 2p_3/2_, respectively. After P doping, all these peaks shift toward higher binding energies, further indicating a decrease in electron density around Co atoms due to P doping.^[^ ^32^, ^33^, ^34^, ^35^ ^]^ A similar binding energy shift is observed in the Se 3d spectra between P‐C/Z@C and C/Z@C (Figure S6a, Supporting Information). Additionally, distinct peaks at 134.3 and 133.6 eV, corresponding to P 2p_1/2_ and P 2p_3/2_ (Figure S6b, Supporting Information), are present in P‐C/Z@C but absent in C/Z@C, confirming the successful doping of P.

Figure 2d displays the morphology of P‐C/Z@C. The SEM image reveals an interconnected nanosheet structure, while the inset TEM image shows that P‐C/Z particles are embedded within the carbon nanosheets. The average size of P‐C/Z@C is 31.86 nm with a size distribution of 10–60 nm (Figure S7, Supporting Information). Compared to the morphology of C/Z@C (Figure S8a,b, Supporting Information), no significant morphological changes are observed after P doping. Therefore, the enhancement in K⁺ storage performance can be attributed to factors other than morphology. The high‐resolution TEM (HRTEM) image of P‐C/Z@C (Figure 2e) reveals two distinct regions with different lattice fringes. Inverse fast Fourier transform (IFFT) analysis was used to determine the lattice spacing and identify the corresponding phases. A lattice spacing of 0.321 nm corresponds to the (111) plane of ZnSe, while a spacing of 0.258 nm corresponds to the (210) plane of CoSe_2_. These regions are attributed to ZnSe and CoSe_2_, respectively, and their interface defines a clear phase boundary, marked by the blue dashed line.^[^ ^30^, ^36^ ^]^ The HRTEM image of C/Z@C is provided in Figure S8c (Supporting Information), where ZnSe (0.327 nm for the (111) plane), CoSe_2_ (0.262 nm for the (210) plane), and the phase boundary are also clearly observed. Notably, the lattice spacings of the (111) plane of ZnSe and the (210) plane of CoSe_2_ in P‐C/Z@C are slightly smaller than those in C/Z@C. This lattice contraction can be ascribed to the substitution of Se atoms by smaller P atoms, providing further evidence of successful P doping into the P‐C/Z@C structure.

To experimentally verify the beneficial effect of the heterophase on P doping, a more detailed analysis was conducted. The XRD patterns, SEM images, and EDS mappings of P‐C@C and P‐Z@C are presented in Figures S9–S11 (Supporting Information), confirming the successful synthesis of both materials. A comparative analysis of the XRD patterns is shown in the enlarged view in Figure 2f, highlighting the differences among P‐C/Z@C vs. C/Z@C, P‐C@C vs. C@C, and P‐Z@C vs. Z@C. For P‐C/Z@C *vs*. C/Z@C, the diffraction peaks of P‐C/Z@C exhibit a noticeable shift toward higher angles, with a Δθ of 0.40°, indicating lattice contraction due to the substitution of Se atoms by smaller P atoms.^[^ ^33^ ^]^ A smaller peak shift (Δθ = 0.04°) is observed in P‐C@C compared to C@C. In contrast, P‐Z@C shows almost no discernible peak shift relative to Z@C. The largest peak shift in P‐C/Z@C suggests a higher degree of P doping, indicating that the heterophase facilitates more effective P doping. To further quantify the P content, ICP‐OES analysis is performed (Figure 2g). The atomic percentages of P in P‐C/Z@C, P‐C@C, and P‐Z@C are found to be 34.0%, 20.8%, and 9.9%, respectively. The highest P content in P‐C/Z@C further confirms that the heterophase plays a critical role in enhancing P doping efficiency. It is important to note that the carbon nanosheets in all selenide composites also serve as potential P‐doping sites. However, since the same carbon source precursor and molar ratio were used across all samples (as detailed in the Experimental Section), the differences in P content can be primarily attributed to the presence or absence of heterophase rather than variations in the carbon matrix itself.

Figure 2h presents the high‐angle annular dark‐field (HAADF) image along with the corresponding elemental mapping of P‐C/Z@C. The distributions of Co and Zn are clearly separated, while both align with the spatial distribution of Se, confirming the formation of a boundary between CoSe_2_ and ZnSe. The doped element P is uniformly distributed across the sample. To further investigate the spatial distribution of P, a line scan was performed along the red dashed line indicated in the HAADF image (Figure 2h). As shown in Figure 2i, the intensity profiles reflect the distribution of each element. At the interface where Co and Zn exhibit high intensities—corresponding to the boundary—the intensity of P also peaks. This indicates that P is predominantly concentrated at the boundaries of CoSe_2_/ZnSe heterophase. The point scan in the different areas further confirms the local high concentration P doping (Figure S12, Supporting Information), where the P contents in the boundaries are higher than that in the bulk areas. These boundaries in the heterophase act as built‐in doping pathways, facilitating enhanced P doping and contributing to the local high‐concentration doping observed in P‐C/Z@C.

**Figure 3:**
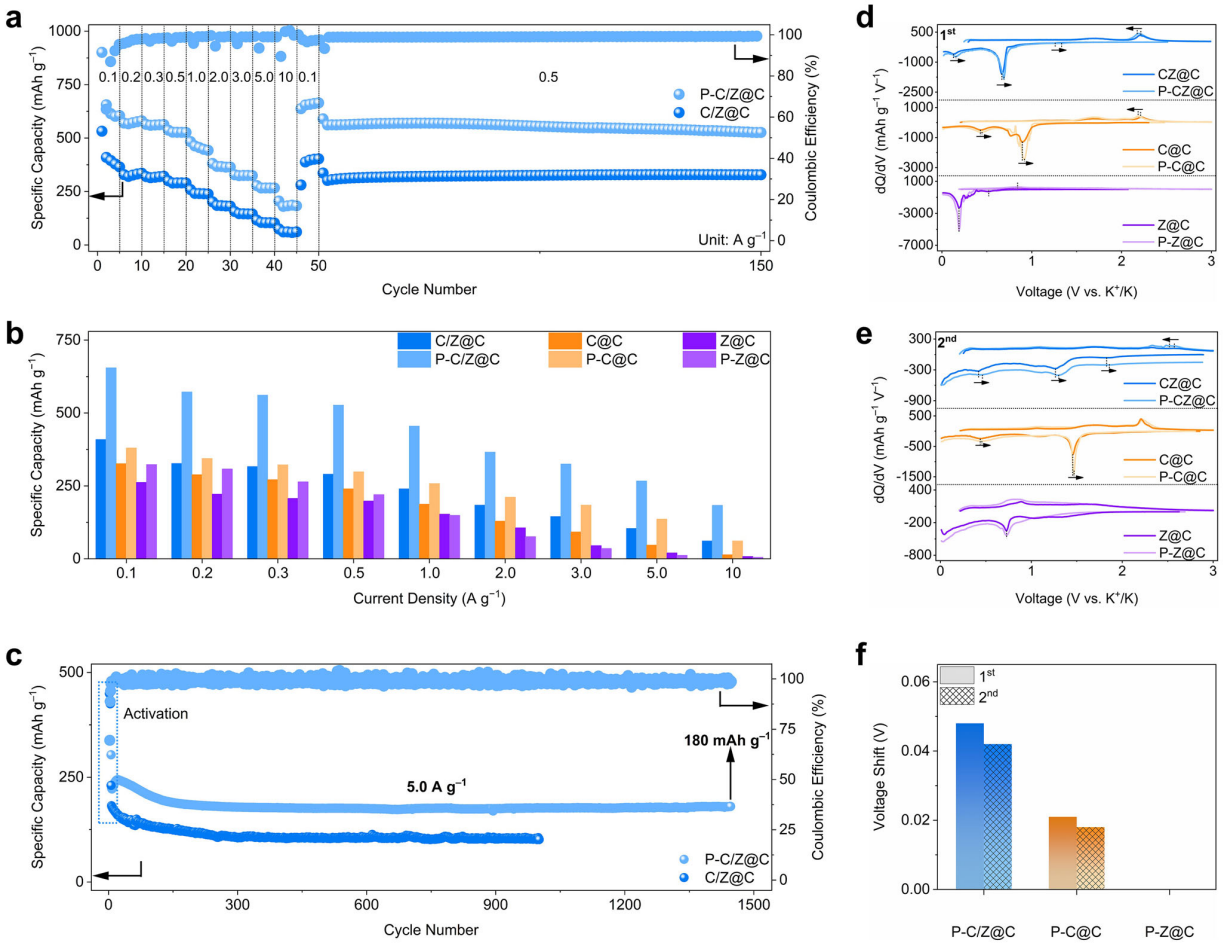
a) Rate performance of P‐C/Z@C and C/Z@C; b) comparison of rate performance; c) cycling performance of P‐C/Z@C and C/Z@C at 5.0 A g^−1^; *dQ*/*dV* curves at the d) 1st cycle and e) 2nd cycle; f) voltage shifts of the reduction peaks at the 1st and 2nd cycle obtained from (d, e).

The K^+^ storage capability was evaluated using CR2032 coin‐type cells. **Figures** **3**a and S13 (Supporting Information) show the rate performance of selenide‐based electrodes before and after P doping. Compared to their undoped counterparts (C/Z@C, C@C, and Z@C), the P‐doped selenides (P‐C/Z@C, P‐C@C, and P‐Z@C) exhibit enhanced rate performance, indicating that P doping effectively promotes K^+^ storage. Among all samples, P‐C/Z@C shows the most outstanding rate performance, delivering specific capacities of 598, 525, 519, 489, 424, 350, 292, 237, and 166 mAh g^−1^ at current densities of 0.1, 0.2, 0.3, 0.5, 1.0, 2.0, 3.0, 5.0, and 10.0 A g^−1^, respectively. When the current density returns to 0.1 A g^−1^, a high reversible capacity of 596 mAh g^−1^ is retained, demonstrating excellent rate capability. Moreover, P‐C/Z@C maintains a stable capacity of 527 mAh g^−1^ at 0.5 A g^−1^ after 150 cycles. In contrast, C/Z@C exhibits much lower rate performance, with capacities of only 338 mAh g^−1^ at 0.1 A g^−1^ and 39 mAh g^−1^ at 10.0 A g^−1^. Compared with other reported conversion‐type anodes, the rate capability of P‐C/Z@C is highly competitive (Figure S14, Supporting Information).

The impact of local high‐concentration doping induced by heterophase engineering is clarified. Figure 3b compares the rate performance of P‐C/Z@C *vs*. C/Z@C, P‐C@C *vs*. C@C, and P‐Z@C *vs*. Z@C. While P doping improves the rate performance in both P‐C@C and P‐Z@C, the enhancement is far less significant than that in P‐C/Z@C. For instance, at 10.0 A g^−1^, the capacity increases from 14 mAh g^−1^ (C@C) to 81 mAh g^−1^ (P‐C@C). In contrast, P‐Z@C shows negligible improvement over Z@C, with capacities of 8 and 6 mAh g^−1^, respectively. Notably, the capacity of P‐C/Z@C at 10.0 A g^−1^ reaches 166 mAh g^−1^, compared to just 39 mAh g^−1^ for C/Z@C. These results confirm that the local high‐concentration P doping enabled by heterophase engineering more effectively improves K^+^ storage capability than in single‐phase selenides with lower doping levels. Additionally, the long‐term cycling performance of P‐C/Z@C and C/Z@C at 5.0 A g^−1^ is shown in Figure 3c. Before cycling at 5.0 A g^−1^, the electrodes are activated at 0.1 A g^−1^ for 5 cycles to achieve a stable electrode interface. After 1400 cycles, P‐C/Z@C maintains a high reversible capacity of 180 mAh g^−1^, outperforming C/Z@C (101 mAh g^−1^). This demonstrates the excellent cycling stability and durability of P‐C/Z@C under high current density, highlighting its promise as a high‐performance anode material for KIBs.

To further validate the beneficial effects of local high‐concentration P doping on K^+^ storage, differential capacity (*dQ*/*dV*) profiles derived from GCD curves (Figures S15–S17, Supporting Information) are presented in Figure 3d,e. As shown in Figure 3d, in the first cycle at 0.1 A g^−1^, the reduction peaks of P‐C/Z@C exhibit a noticeable shift toward higher voltages compared to C/Z@C. This shift indicates reduced voltage polarization and enhanced K^+^ storage kinetics. A similar shift is also observed between P‐C@C and C@C. However, no apparent shift is observed between P‐Z@C and Z@C, suggesting limited kinetic enhancement in the absence of heterophase. In the second cycle (Figure 3e), the voltage shifts between P‐C/Z@C *vs*. C/Z@C and P‐C@C *vs*. C@C persist, indicating that the effect of P doping remains active during cycling. Again, no shift is seen between P‐Z@C and Z@C. In the positive scan, the P‐C/Z@C also shows the higher voltage shift to the low potential direction in the first cycle (0.04 V) and second cycle (0.05 V), implying the enhanced K^+^ storage kinetics at the highest degree. A detailed comparison of the voltage shifts in the reduction peaks is provided in Figure 3f. P‐C/Z@C shows the most pronounced voltage shifts, with 0.048 V in the first cycle and 0.042 V in the second cycle. In contrast, P‐C@C *vs*. C@C exhibits smaller shifts of 0.021 and 0.018 V in the first and second cycles, respectively, while P‐Z@C *vs*. Z@C shows no measurable shift in either cycle. These results clearly demonstrate that the heterophase in P‐C/Z@C enables a local high‐concentration doping with a higher P doping content, which in turn significantly enhances both the K⁺ storage capacity and kinetics. Compared to P‐C@C and P‐Z@C—where the absence of heterophase limits the doping level and performance improvement—P‐C/Z@C exhibits superior electrochemical behavior, confirming the critical role of the heterophase in enabling the local high‐concentration P doping and high‐performance K^+^ storage.

**Figure 4:**
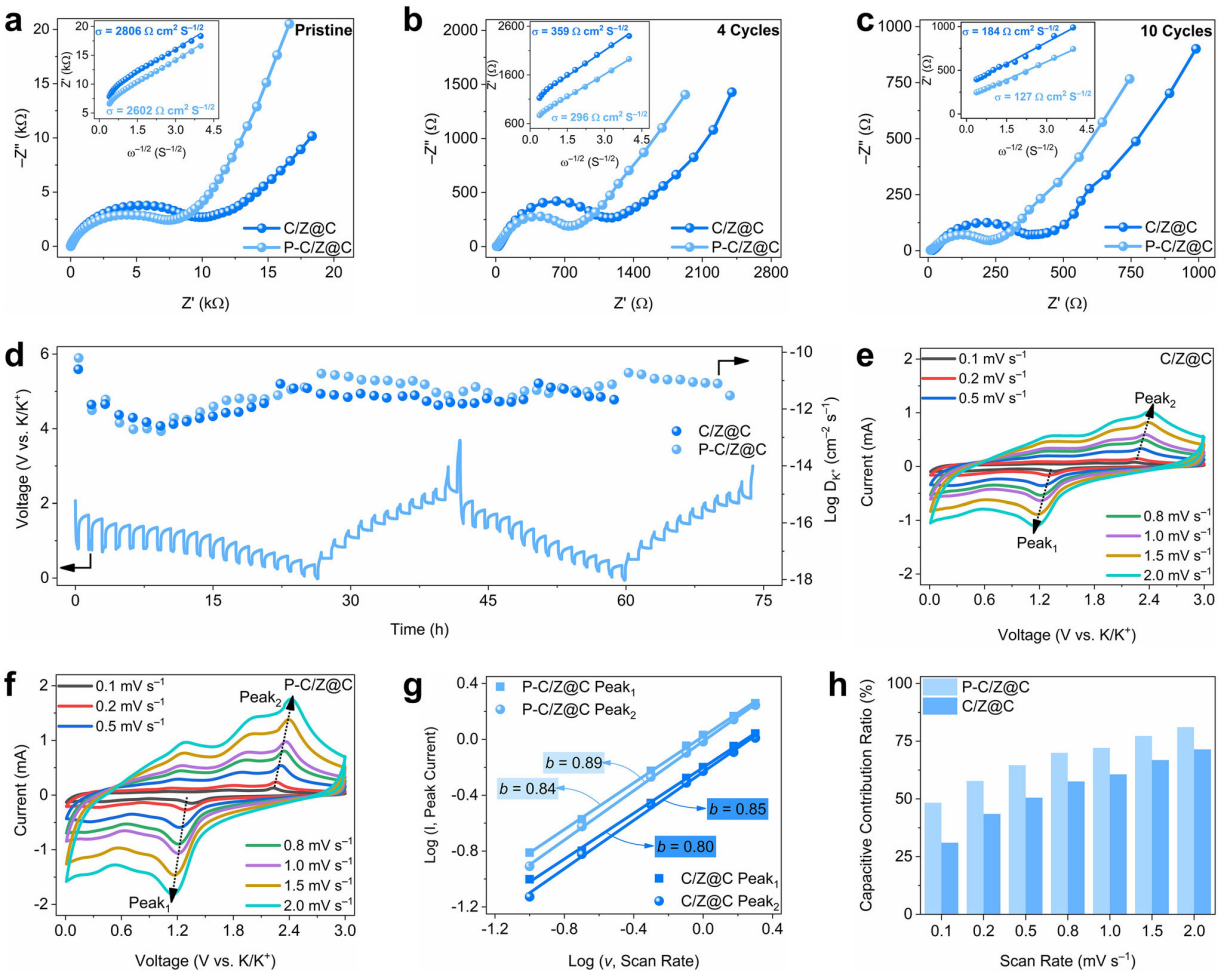
EIS spectra and corresponding Z' *vs*. ω^−1/2^ relationships at low‐frequency regions of P‐C/Z@C and C/Z@C for the a) pristine, b) 4 cycles, and c) 10 cycles; d) $D_{K^{+}}$ of P‐C/Z@C and C/Z@C, and GITT curves of P‐C/Z@C; CV curves at scan rates varying from 0.1 to 2.0 mV s^−1^ of e) C/Z@C and f) P‐C/Z@C; g) relationships between peak currents and scan rates of P‐C/Z@C and C/Z@C; h) capacitive contribution ratio of P‐C/Z@C and C/Z@C at various scan rates.

The positive effect of the local high‐concentration P doping on K^+^ storage is verified by investigating the electrochemical kinetics through electrochemical impedance spectroscopy (EIS). In the EIS spectra, the semicircle observed in the high‐to‐medium frequency region corresponds to the charge transfer resistance (*R*_*ct*_), while the inclined line in the low‐frequency region represents the Warburg impedance (*R*_*w*_), which is associated with ion diffusion.^[^ ^12^, ^37^, ^38^, ^39^ ^]^ As shown in **Figure** **4**a, the EIS spectra of the pristine P‐C/Z@C and C/Z@C electrodes reveal that P‐C/Z@C exhibits a significantly lower *R*_*ct*_ than C/Z@C, indicating faster charge transfer at the electrode–electrolyte interface. Using the equation *Z'* = *R*_*s*_ + *R*_*ct*_ + *σω* ^−1/2^, the Warburg coefficient (*σ*), which reflects ion diffusion resistance, is calculated from the linear fitting of *Z'* versus *ω* ^−1/2^.^[^ ^12^ ^]^ For the pristine electrodes, P‐C/Z@C exhibits a lower *σ* value of 2602 Ω cm s^−1/2^ compared to 2806 Ω cm s^−1/2^ for C/Z@C (inset of Figure 4a), confirming enhanced K⁺ diffusion in the P‐doped material. Furthermore, EIS measurements after 4 cycles (Figure 4b) and 10 cycles (Figure 4c) show that P‐C/Z@C consistently maintains a lower *R*_*ct*_ and a smaller *σ* value than C/Z@C. This indicates that the improved charge transfer and ion diffusion kinetics persist during cycling. These results demonstrate that P doping at the phase boundary continuously enhances K⁺ storage kinetics, likely because anion doping in conversion‐type anode materials remains stable throughout repeated cycling.^[^ ^13^ ^]^

The galvanostatic intermittent titration technique (GITT) is employed to evaluate the potassium‐ion diffusion coefficient ($D_{K^{+}}$) of P‐C/Z@C and C/Z@C, further confirming the positive effect of local high‐concentration P doping on K⁺ storage kinetics. The $D_{K^{+}}$ Values are calculated using the following equation: $D_{K^{+}}=\;\frac{4}{\Pi \tau }\;{\left(\frac{m_{B}\;V_{m}}{M_{B}\;A}\right)}^{2}\;{\left(\frac{\Delta E_{s}}{\Delta E_{\tau }}\right)}^{2}$, where *m_B_*, *V_m_*, and *M_B_* represent the mass, molar volume, and molar mass of active materials, respectively; *A*, *ΔE_s_*, and *ΔE_τ_* represent the contact area between electrode and electrolyte, the voltage variation during two steady open‐circuit potentials, and voltage variation in the rest time, respectively.^[^ ^13^, ^33^ ^]^ Figures 4d and S18 (Supporting Information) display the GITT curves of P‐C/Z@C and C/Z@C, with charge/discharge performed at 0.1 A g^−1^ for 20 min followed by a 60 min rest period. Local‐enlarged GITT curves are provided in Figure S19 (Supporting Information). Compared to C/Z@C, P‐C/Z@C consistently exhibits higher $D_{K^{+}}$ values during both discharge and charge processes in the first and second cycles, indicating faster K^+^ diffusion and improved ion transport kinetics. The P‐C/Z@C exhibits a higher average $D_{K^{+}}$ of 6.98 × 10^−12^ cm^2^ s^−1^ than that of C/Z@C (3.28 × 10^−12^ cm^2^ s^−1^) in the first and second cycles. These findings clearly demonstrate that local high‐concentration P doping enabled by heterophase effectively enhances K^+^ diffusion, thereby improving K^+^ storage kinetics. Furthermore, the sustained enhancement in $D_{K^{+}}$ over multiple cycles suggests that the P‐doped structure remains stable even after conversion reactions—an observation that aligns well with the EIS results discussed earlier.

Cyclic voltammetry (CV) is conducted to investigate the K^+^ storage behavior and kinetics of P‐C/Z@C and C/Z@C. The CV curves, recorded in the voltage range of 0.01–3.0 V at a scan rate of 0.1 mV s^−1^, are shown in Figure S20 (Supporting Information). After the first cycle, the reduction peaks in both materials shift toward higher voltages, and the peak near 0.1 V disappears. This behavior is attributed to the activation process and the formation of a solid electrolyte interphase (SEI) layer. In the subsequent cycles, the CV curves for both P‐C/Z@C and C/Z@C overlap well, indicating excellent electrochemical reversibility and cycling stability. For P‐C/Z@C, the reduction peak at ≈1.3 V corresponds to the intercalation of K^+^ into CoSe_2_ to form K_x_CoSe_2_.^[^ ^40^, ^41^ ^]^ The peaks at ~0.8 and 0.4 V are associated with the conversion reactions of ZnSe and K_x_CoSe_2_, respectively, leading to the formation of K_2_Se, Zn, and Co.^[^ ^42^, ^43^ ^]^ The low‐potential peak ≈0.01 V is attributed to the alloying reaction between Zn and K^+^ to form KZn_13_.^[^ ^43^ ^]^ During the anodic scan, the oxidation peaks at ~1.1 and ~1.8 V correspond to the reverse conversion reactions, regenerating K_x_CoSe_2_ and ZnSe, respectively. The peak at ~2.2 V is attributed to the deintercalation of K^+^ from K_x_CoSe_2_.^[^ ^36^ ^]^ The similarity in peak positions between C/Z@C and P‐C/Z@C indicates that the introduction of P at the boundary of heterophase does not alter the underlying K^+^ storage mechanism. However, the current intensities of both the reduction and oxidation peaks are significantly higher in P‐C/Z@C than those in C/Z@C, reflecting enhanced K^+^ storage capacity and faster electrochemical kinetics. These improvements are attributed to the beneficial effects of local high‐concentration P doping, which promotes better conductivity and ion transport.

Figure 4e,f present the CV curves of P‐C/Z@C and C/Z@C at various scan rates ranging from 0.1 to 2.0 mV s^−1^. The relationship between peak current (*I*) and scan rate (*v*) follows a power‐law expression: *I* = *a v^b^*, where the value of *b* provides insight into the charge storage behavior. By taking the logarithm of both sides and applying linear fitting, the *b* value can be determined. A *b* value of 0.5 typically indicates a diffusion‐controlled Faradaic process, while a value of 1.0 suggests a surface‐controlled capacitive (double‐layer or pseudocapacitive) process.^[^ ^30^, ^44^, ^45^ ^]^ The peak currents of *Peak_1_* and *Peak_2_*, marked in Figure 4e,f, are analyzed accordingly. As shown in Figure 4g, P‐C/Z@C exhibits higher *b* values (*b_1_* = 0.84 and *b_2_* = 0.89) compared to those of C/Z@C (*b_1_* = 0.80 and *b_2_* = 0.85), indicating a greater contribution from capacitive processes and more rapid charge transfer kinetics in the P‐C/Z@C. To further quantify the capacitive contribution, the current response at a given voltage *i*(V) is separated into capacitive (*k_1_ V*) and diffusion‐controlled (*k_2_ V^1/2^*) components using the equation *i*(V) = *k_1_ V* + *k_2_ V^1/2^*.^[^ ^46^, ^47^, ^48^ ^]^ At a scan rate of 2.0 mV s^−1^, the capacitive contribution ratios for P‐C/Z@C and C/Z@C were calculated to be 81% and 71%, respectively (Figure S21, Supporting Information), indicating a more prominent capacitive behavior in P‐C/Z@C. Figure 4h summarizes the capacitive contributions across various scan rates, consistently showing higher capacitive ratios for P‐C/Z@C than for C/Z@C. A greater capacitive contribution signifies more efficient electron and K^+^ transport, reflecting superior K^+^ storage kinetics—an essential characteristic for achieving excellent rate performance in the anodes of KIBs.

The comprehensive analysis of K^+^ storage kinetics reveals that the local high‐concentration P doping enabled by heterophase engineering effectively reduces charge transfer resistance and enhances K⁺ diffusion throughout the cycling process. These improvements can be attributed to the strengthened electric field and the weakened bound strength introduced by P doping in the boundary of heterophase, which collectively facilitate both intercalation and conversion reactions. Furthermore, P doping modulates the K^+^ storage behavior by increasing the contribution from capacitive processes, thereby significantly enhancing the rate capability.

**Figure 5:**
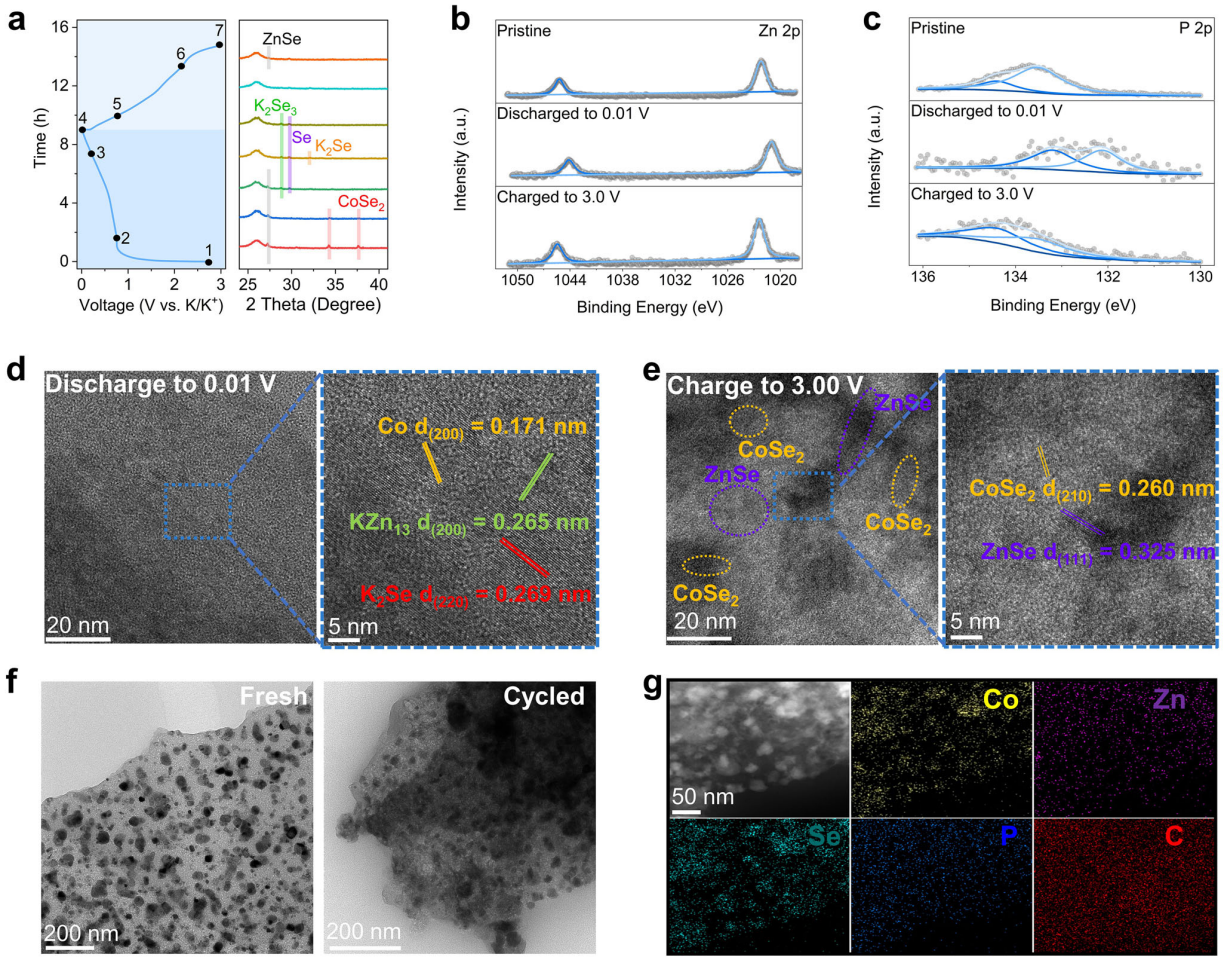
a) GCD curves and corresponding ex situ XRD patterns of P‐C/Z@C; ex situ high‐resolution b) Zn 2p and c) P 2p XPS spectra of P‐C/Z@C; ex situ TEM and HRTEM image of P‐C/Z@C electrodes after d) discharge to 0.01 V and e) charge to 3.0 V; f) comparison of HRTEM image between fresh and cycled P‐C/Z@C; (g) HAADF image and element mappings of cycled P‐C/Z@C.

The composition and structure of P‐C/Z@C undergo significant changes during the cycling process. Determining whether P doping and the phase boundary are retained after cycling is essential to elucidate the mechanism behind the enhanced K⁺ storage resulting from the local high‐concentration P doping. To investigate this, a series of exsitu characterizations is conducted. **Figure** **5**a presents the GCD curves at 0.1 A g^−1^ along with the corresponding ex situ XRD patterns. Upon discharging to 1.2 V (*Point 2*), a slight shift of the CoSe_2_ diffraction peaks toward lower angles is observed, indicating K⁺ intercalation. At 0.3 V (*Point 3*), the ZnSe peak intensity decreases and the CoSe_2_ peaks vanish, accompanied by the emergence of new peaks corresponding to Se (29.7°) and K_2_Se_3_ (28.8°), implying conversion reactions. Upon further discharge to 0.01 V (*Point 4*), the ZnSe peaks disappear completely, while peaks of K_2_Se (32.1°) appear.^[^ ^42^, ^49^, ^50^, ^51^ ^]^ When the electrode is charged to 0.8 V (*Point 5*), the K_2_Se peak disappears, corresponding to the reverse conversion reaction. After charging to 1.8 V (*Point 6*), the peaks of K_2_Se_3_ and Se also vanish, indicating the completion of the reverse reaction. Upon full charging to 3.0 V (*Point 7*), the ZnSe peaks reappear, while the CoSe_2_ peaks remain absent.^[^ ^37^, ^39^, ^40^, ^41^ ^]^ This absence is likely due to significant volume changes during cycling, which induce particle pulverization and result in poor crystallinity of CoSe_2_.^[^ ^51^ ^]^ These results indicate a difference in reversibility between ZnSe and CoSe_2_ during K⁺ uptake and release. Therefore, it is inferred that the phase boundary between ZnSe and CoSe_2_ may be disrupted after the conversion and reverse‐conversion processes. This hypothesis will be further validated through ex situ TEM analysis in the following section.

Ex situ XPS analysis is performed to investigate the valence state evolution of elements in P‐C/Z@C during the discharge–charge process. Figure 5b shows the ex situ Zn 2p spectra. For the pristine electrode, the Zn 2p_1/2_ and Zn 2p_3/2_ peaks are located at 1045.3 and 1022.2 eV, respectively. Upon discharging to 0.01 V, both peaks shift to lower binding energies (Zn 2p_1/2_: 1044.2 eV, Zn 2p_3/2_: 1021.0 eV), indicating the reduction of Zn^2^⁺ and the formation of the Zn‐based alloy KZn_13_. After recharging to 3.0 V, the peaks shift back to higher binding energies (Zn 2p_1/2_: 1045.4 eV, Zn 2p_3/2_: 1022.3 eV), suggesting dealloying of KZn_13_ and reoxidation of Zn to Zn^2+^ in ZnSe.^[^ ^52^ ^]^ A similar trend is observed in the Co 2p spectra (Figure S22, Supporting Information), corresponding to the reduction during discharge and its reoxidation during charge.^[^ ^53^ ^]^ Furthermore, ex situ P 2p spectra are collected to assess the evolution of the dopant P at the phase boundary. As shown in Figure 5c, the P 2p_1/2_ and P 2p_3/2_ peaks for the pristine electrode are located at 134.4 eV and 133.5 eV, respectively. Upon discharging to 0.01 V, these peaks shift to lower binding energies (P 2p_1/2_: 133.2 eV, P 2p_3/2_: 132.2 eV), and return to their original positions after charging to 3.0 V, indicating a reversible change in the local chemical environment of P during cycling.^[^ ^13^ ^]^ These results confirm that P remains incorporated as a dopant, consistent with previous reports on Se‐doped In_2_S_3_ and Cu‐doped Bi_2_S_3_ used as KIB anode materials.^[^ ^12^, ^13^ ^]^

Ex situ TEM analysis is conducted to further investigate the structural evolution of P‐C/Z@C during cycling. Figure 5d presents the TEM image of P‐C/Z@C after full discharge to 0.01 V and the corresponding HRTEM image of the selected region. The measured lattice spacings of 0.171, 0.265, and 0.269 nm can be assigned to the (200) plane of metallic Co, the (200) plane of KZn_13_, and the (220) plane of K_2_Se, respectively.^[^ ^41^, ^43^, ^49^ ^]^ The inverse fast Fourier transform (IFFT) images and associated lattice spacings are provided in Figure S23a–c (Supporting Information). Notably, the lattice spacing of K_2_Se appears smaller than typical values reported for other selenide systems, which can be attributed to P doping into the K_2_Se lattice—consistent with previous observations in Se‐doped In_2_S_3_.^[^ ^13^, ^50^, ^54^, ^55^ ^]^ Figure 5e shows the TEM image of the electrode after recharging to 3.00 V. The boundary between CoSe_2_ and ZnSe is no longer observed, with the two phases becoming spatially separated. As an example, the region marked in Figure 5e reveals lattice spacings of 0.260 nm and 0.325 nm, corresponding to the (210) plane of CoSe_2_ and the (111) plane of ZnSe, respectively, and IFFT images and corresponding lattice spacings are provided in Figure S24a,b (Supporting Information).^[^ ^41^, ^43^, ^49^ ^]^ The disappearance of the boundary is attributed to particle fragmentation caused by severe volume changes and the limited structural reversibility of CoSe_2_, as discussed in the ex situ XRD analysis. Figure 5f compares TEM images of P‐C/Z@C before and after cycling. In the fresh electrode, distinct P‐C/Z nanoparticles embedded in the carbon matrix are clearly visible. After cycling, the nanoparticles become less defined and appear smaller, confirming that significant volume fluctuations lead to particle fragmentation and subsequent boundary loss.^[^ ^56^ ^]^ Further structural evidence is provided in Figure 5g, which displays the HAADF image and corresponding elemental maps of the cycled P‐C/Z@C electrode. Co and Zn are distributed separately due to particle fragmentation and different reversibility of CoSe_2_ and ZnSe, differing significantly from the well‐organized heterophase in the pristine material (Figure 2h). Nevertheless, the element P is distributed in the charged products, suggesting that P continues to be re‐doped into both CoSe_2_ and ZnSe phases after cycling.

Based on these observations, the structural evolution of P‐C/Z@C is illustrated in Scheme S1 (Supporting Information). Initially, the doping element P is locally concentrated at the boundary of the heterophase between CoSe_2_ and ZnSe (P–CoSe_2_/ZnSe). Upon cycling, the boundary is disrupted, and P remains doped evenly in the separated CoSe_2_ and ZnSe (P–CoSe_2_ + P–ZnSe). This persistent P doping continues to contribute to electrochemical performance in subsequent cycles.

**Figure 6:**
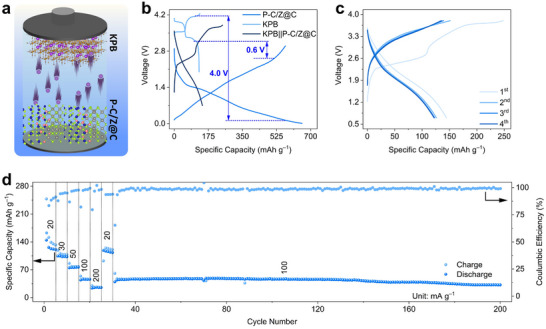
a) Full‐battery configuration of KPB||P‐C/Z@C; b) GCD curves of KPB and P‐C/Z@C to fix the working voltage range of full battery; c) GCD curves of KPB||P‐C/Z@C; d) rate and cycling performance of KPB||P‐C/Z@C.

The practical applicability of P‐C/Z@C is demonstrated using a full battery, as illustrated in **Figure** **6**a, with potassium Prussian blue (KPB) serving as the cathode. The XRD pattern and SEM image indicate the successful synthesis of KPB (Figure S25, Supporting Information). Figure 6b shows the GCD curves of both KPB and P‐C/Z@C, which are used to determine the appropriate voltage window for the KPB||P‐C/Z@C full battery. During the charging process, K^+^ ions are extracted from KPB and stored in P‐C/Z@C. The upper voltage limit (4.0 V) is defined by the difference between the KPB charge plateau (≈4.1 V) and the discharge plateau of P‐C/Z@C (≈0.1 V). Similarly, the lower voltage limit (0.6 V) is determined by the potential gap between the discharge plateau of KPB (≈3.2 V) and the charge plateau of P‐C/Z@C (≈2.6 V). Thus, the operating voltage range of the KPB||P‐C/Z@C full battery is 0.6–4.0 V.^[^ ^13^, ^28^ ^]^ All current densities and specific capacities are calculated based on the mass of the KPB cathode. As shown in Figure 6c, the full battery delivers a high initial discharge capacity of 149 mAh g^−1^ at 20 mA g^−1^. In subsequent cycles, the charge and discharge profiles remain consistent, and a reversible capacity of 124 mAh g^−1^ is retained, highlighting the excellent reversibility and stability of the KPB||P‐C/Z@C full battery. The rate and cycling performance of the full battery are summarized in Figure 6d. KPB||P‐C/Z@C exhibits excellent rate capability, delivering capacities of 123, 105, 75, 46, and 26 mAh g^−1^ at current densities of 20, 30, 50, 100, and 200 mA g^−1^, respectively. Notably, when the current density is returned to 20 mA g^−1^, the capacity quickly recovers to 118 mAh g^−1^, corresponding to an energy density of 218 Wh kg^−1^, confirming the good rate reversibility of the full battery. Furthermore, when cycled at 100 mA g^−1^, the full battery maintains a reversible capacity of 22.3 mAh g^−1^ even after 420 cycles, demonstrating outstanding long‐term cycling stability. Overall, the superior electrochemical performance of the KPB||P‐C/Z@C full battery underscores the critical role of the boundary of heterophase as an intrinsic doping channel that enables local high‐concentration P doping. These findings highlight the promise of this design strategy for next‐generation LSESSs.

## 3 Conclusion

In summary, the boundary in the CoSe_2_/ZnSe heterophase is demonstrated to act as a built‐in doping tunnel, enabling local high‐conentration P doping due to its low diffusion barrier and low formation energy during the doping process. The elevated P doping content at the boundary of heterophase intensifies the intrinsic electric field to accelerate K^+^ intercalation and weakens the metal–Se bond strength to promote the conversion reaction. These synergistic effects contribute to significantly improved K^+^ storage kinetics. As a result, the P‐C/Z@C composite exhibits outstanding rate performance (598 mAh g^−1^ at 0.1 A g^−1^, 166 mAh g^−1^ at 10.0 A g^−1^) and long‐term cycling stability (180 mAh g^−1^ at 5.0 A g^−1^ after 1400 cycles). Moreover, structural evolution analysis reveals that the initial local high‐concentration P doping in P‐C/Z@C transitions into separate P doping in CoSe_2_ and ZnSe due to volume expansion during cycling, leading to the disappearance of the boundary. The practical applicability of P‐C/Z@C is further validated in a full‐cell configuration, delivering a high‐power energy density of 218 Wh kg^−1^. This work not only proposes a novel strategy of local high‐concentration doping enabled by heterophase engineering in conversion‐type anodes but also offers deep insight into the dynamic structural evolution of heterophase materials, thereby providing valuable guidance for the design of high‐energy‐density anodes for KIBs.

## 4 Experimental Section

### Synthesis of C/Z@C, C@C, Z@C

Initially, 1.5 g of polyvinylpyrrolidone (PVP, K88‐K96, Aladin) was dissolved in 50 mL of deionized (DI) water under continuous stirring. Subsequently, 0.75 g of zinc nitrate hexahydrate (Zn(NO_3_)_2_·6H_2_O, 99.99%, Aladin) and 0.75 g of cobalt nitrate hexahydrate (Co(NO_3_)_2_·6H_2_O, 99.99%, Aladin) were added to the PVP solution. The mixture was stirred until a clear and homogeneous solution was obtained. The resulting solution was then frozen and subjected to freeze‐drying to yield a solid precursor. This precursor was calcined in a tube furnace at 700 °C for 2 h under an Ar atmosphere with a heating rate of 4 °C min^−1^, followed by natural cooling to room temperature. The calcined product was thoroughly mixed with selenium powder at a mass ratio of 1:2, followed by a second calcination at 500 °C for 4 h under an Ar atmosphere with a heating rate of 2 °C min^−1^. After natural cooling to room temperature, the final C/Z@C composite was obtained.

The preparation of C@C followed the same procedure as C/Z@C, except that only 1.5 g of Co(NO_3_)_2_·6H_2_O was added to the PVP solution without Zn(NO_3_)_2_·6H_2_O. Similarly, the synthesis of Z@C was carried out by adding 1.5 g of Zn(NO_3_)_2_·6H_2_O alone, excluding Co(NO_3_)_2_·6H_2_O, following the same steps described above.

### Synthesis of P‐C/Z@C, P‐C@C, P‐Z@C

A total of 0.05 g of C/Z@C and 0.2 g of sodium hypophosphite (NaH_2_PO_2_, 98‐99%, Aladin) were placed at the downstream and upstream positions, respectively, of a horizontal tube furnace. The system was then heated to 350 °C for 3 h at a heating rate of 2 °C min^−1^ under an Ar atmosphere. After natural cooling to room temperature, the P‐C/Z@C was obtained.

P‐C@C and P‐Z@C were synthesized following the same procedure as P‐C/Z@C, except that C/Z@C was replaced with C@C or Z@C, respectively.

### Synthesis of KPB

A homogeneous solution was prepared by dissolving 2.5 mmol of K_4_Fe(CN)₆·3H_2_O, 5.0 mmol of potassium citrate, 7.50 g of KCl, and 0.25 g of ascorbic acid in 50 mL of deionized (DI) water. Subsequently, a solution containing 2.5 mmol of FeCl_2_·4H_2_O in 50 mL of ethanol was slowly added to the above mixture. The resulting solution was stirred at 60 °C for 6 h under a continuous N_2_ purge. The resulting precipitate was collected by centrifugation, washed thoroughly with DI water and ethanol at least 3 times, and then vacuum‐dried at 100 °C for 48 h to yield the final KPB product.

### Characterization

XRD patterns were recorded using a Haoyuan DX‐2700BH diffractometer with Cu Kα radiation (λ = 1.5406 Å). SEM images and EDS data were obtained using a FEI Sirion 200 microscope operated at an accelerating voltage of 20 kV. TEM, HRTEM, and elemental mappings were conducted on a Thermo Scientific Talos F200X microscope at an accelerating voltage of 200 kV. ICP‐OES measurements were performed using a Thermo Scientific iCAP PRO spectrometer to determine the atomic percentages of P in P‐C/Z@C, P‐C@C, and P‐Z@C. XPS spectra were acquired using a Thermo Scientific K‐Alpha spectrometer equipped with Al Kα radiation.

### Electrochemical Tests

CR2032 coin‐type cells were assembled in an argon‐filled glove box to evaluate the electrochemical performance of both half‐cells and full batteries. The working electrode slurry was prepared by mixing the active materials (P‐C/Z@C, P‐C@C, P‐Z@C, C/Z@C, C@C, and Z@C), carbon black, and sodium carboxymethyl cellulose (CMC‐Na) in DI water at a mass ratio of 7:2:1 to form a homogeneous slurry. This slurry was uniformly coated onto copper foil using the doctor blade method and subsequently dried under vacuum at 100 °C for 24 h. The dried copper foil was then punched into circular electrodes (10 mm in diameter) with a mass loading of ≈1.4 mg cm^−2^. Potassium metal was used as both the counter and reference electrode. For both half‐cells and full batteries, a 4 m KFSI in DME was used as the electrolyte, and a glass fiber separator (Whatman GF/D) was employed. CV measurements were conducted using a CHI 660e electrochemical workstation. EIS was performed on a Bio‐Logic SP‐150 workstation over a frequency range of 0.01 Hz to 100 kHz. GCD and GITT measurements were carried out using a Neware CT‐4008Tn battery testing system. For full battery assembly, the cathode was prepared by mixing KPB, carbon black, and polyvinylidene fluoride (PVDF) in N‐methyl‐2‐pyrrolidone (NMP) at a weight ratio of 8:1:1 to form a uniform slurry. This was coated onto aluminum foil and dried under vacuum at 120 °C for 24 h. The resulting foil was punched into discs (10 mm in diameter). The mass ratio of KPB to P‐C/Z@C in the full battery was 1:3.

### Theoretical Simulations

First‐principles calculations were performed using the Vienna Ab initio Simulation Package (VASP) based on density functional theory (DFT) with the projector augmented wave (PAW) method. The Perdew–Burke–Ernzerhof (PBE) functional within the generalized gradient approximation (GGA) was employed to describe the exchange–correlation interactions. A plane‐wave cutoff energy of 500 eV and a k‐point mesh of 3 × 3 × 1 were used. Structural optimization was conducted with a force convergence criterion of 0.005 eV Å^−1^. To avoid interlayer interactions, a vacuum spacing of more than 15 Å was applied along the c‐axis. The nudged elastic band (NEB) method was used to calculate the P diffusion energy barriers. In addition, the electron localization function (ELF) was computed for CoSe_2_, ZnSe, P‐doped CoSe_2_, P‐doped ZnSe, and P‐doped CoSe_2_/ZnSe to analyze the nature of chemical bonding. ELF analysis, widely used to visualize electron distribution and bonding strength, provides insights into the bonding characteristics between neighboring atoms. The defect formation energy of P doping is calculated by the equation: E_df_ = E_P‐doped selenides_ – E_selenides_ – E_P_, where the E_df_, E_P‐doped selenides_, E_selenides_, and E_P_ correspond to the defect formation energy, the free energy of P‐doped selenides, the free energy of selenides without P doping, and the free energy of P, respectively.

## Conflict of Interest

The authors declare no conflict of interest.

## Supporting information



Supporting Information

## Data Availability

The data that support the findings of this study are available from the corresponding author upon reasonable request.
