# Supplementary material for: Heterophase Engineering Creates a Built‐in Highway to Achieve Local High‐Concentration Phosphorus Doping for Robust K‐Ion Storage
Source: Adv Sci (Weinh). 2025 Nov 5;13(4):e18687. doi: 10.1002/advs.202518687 (PMC12822424; doi:10.1002/advs.202518687)
Supplement: Supplementary file 1 — Supporting Information [file ADVS-13-e18687-s001.docx]

**Supporting Information**

**Local High-Concentration Phosphorus Doping Enabled by Heterophase Engineering for Robust K-Ion Storage**

Dawei Sha^1,2,#^, Yurong You^2,#^, Yuan Zhang^2^, Long Pan^2,*^, ZhengMing Sun^2,*^

1 Institute of Technology for Carbon Neutralization, College of Electrical, Energy and Power Engineering, Yangzhou University, Yangzhou, Jiangsu, China

2 Key Laboratory of Advanced Metallic Materials of Jiangsu Province, School of Materials Science and Engineering, Southeast University, Nanjing, Jiangsu, China

# These authors contributed equally to this work.

* Corresponding Authors: zmsun@seu.edu.cn, [panlong@seu.edu.cn](mailto:panlong@seu.edu.cn)





**Figure S1** (a) The comparison of P doping content between our work and other reported P doped TMCs^[S1-S12]^; (b) the comparison of improvement ratio of specific capacity at high-rate current densities between our work and other reported doped TMCs^[S13-S22]^.





**Figure S2** P diffusion pathways in CoSe_2_ and ZnSe.





**Figure S3** The atomic structure of (a) P-CoSe_2_ (b) and P-ZnSe.

**Table S1** Free energies of P, P-doped selenides, and undoped selenides

| Smaple | P | P-CoSe_2_/ZnSe | CoSe_2_/ZnSe | P-CoSe_2_ | CoSe_2_ | P-ZnSe | ZnSe |
| --- | --- | --- | --- | --- | --- | --- | --- |
| Energy (eV) | –0.18 | –650.08 | –644.16 | –422.69 | –420.88 | –215.01 | –215.18 |


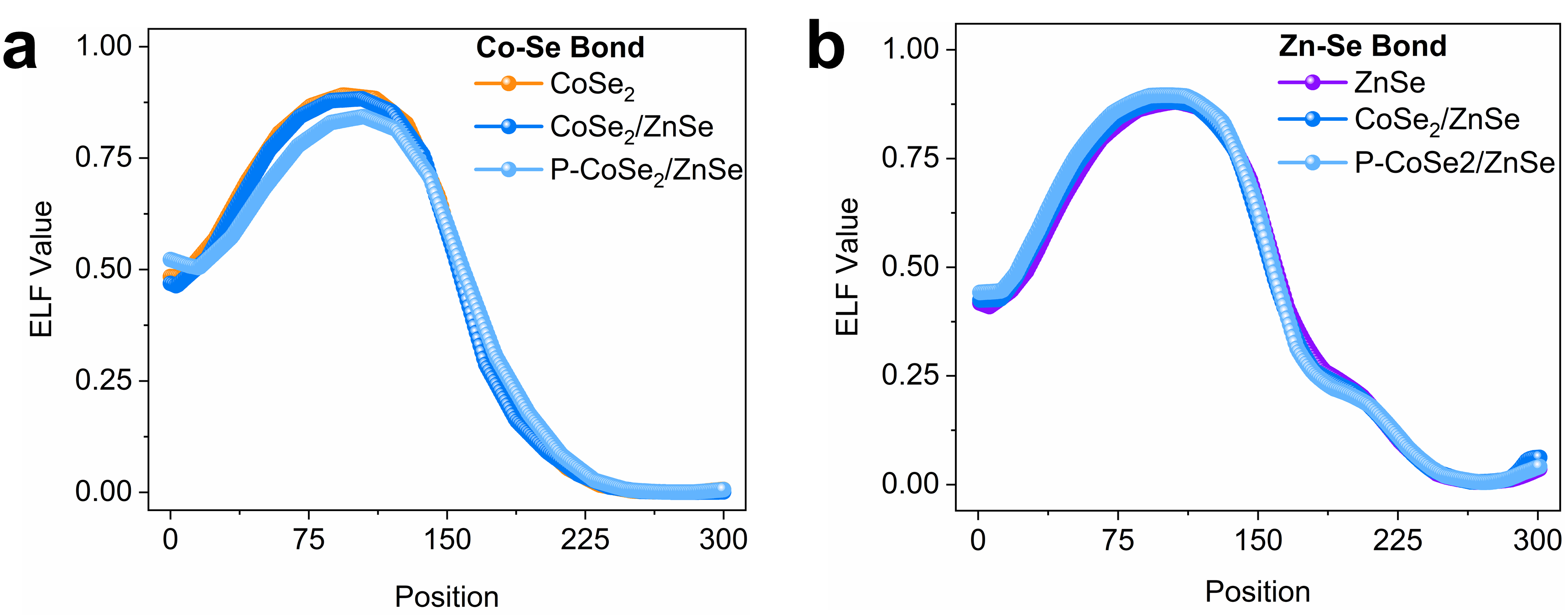


**Figure S4** ELF value plots of (a) Co-Se and (b) Zn-Se bond in CoSe_2_@C, ZnSe@C, and P-CoSe_2_/ZnSe@C.


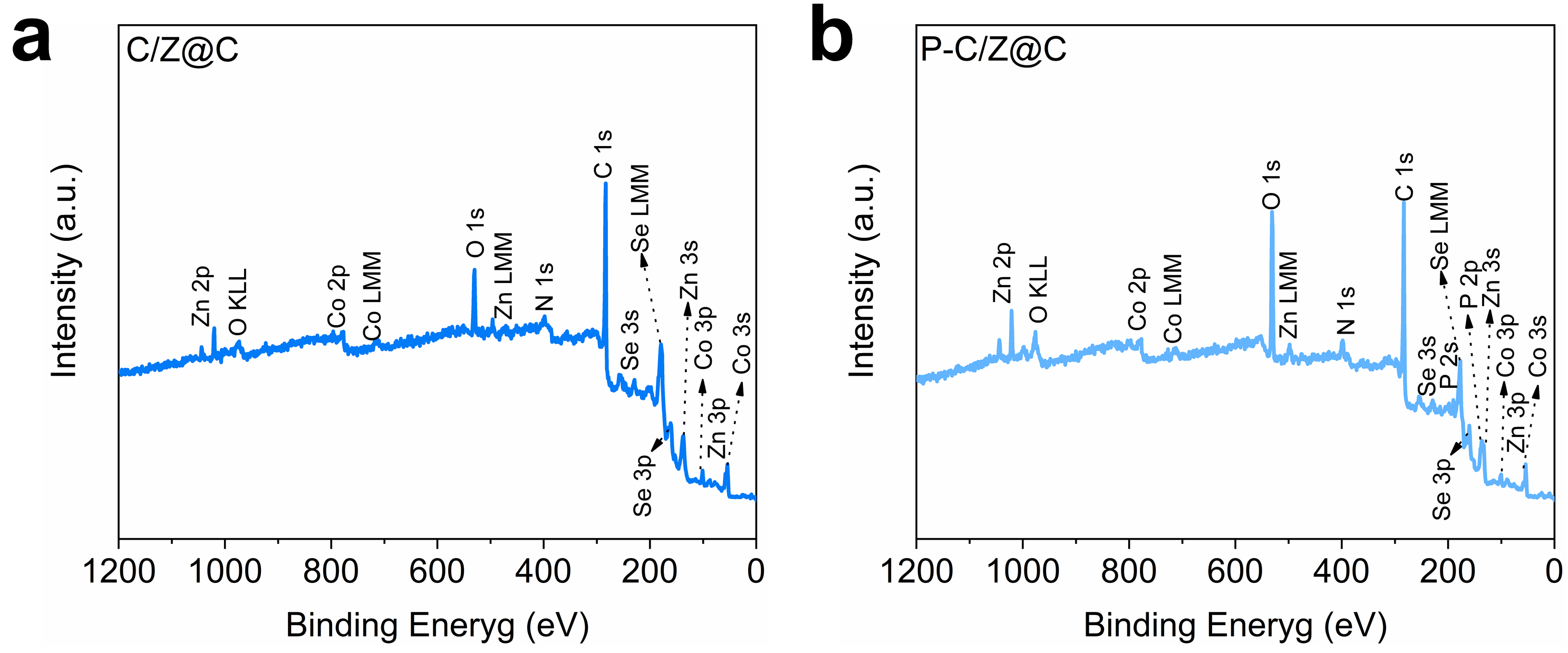


**Figure S5** XPS survey scans of (a) C/Z@C and (b) P-/Z@C.





**Figure S6** High-resolution XPS spectra of (a) Se 3d and (b) P 2p for P-C/Z@C and C/Z@C


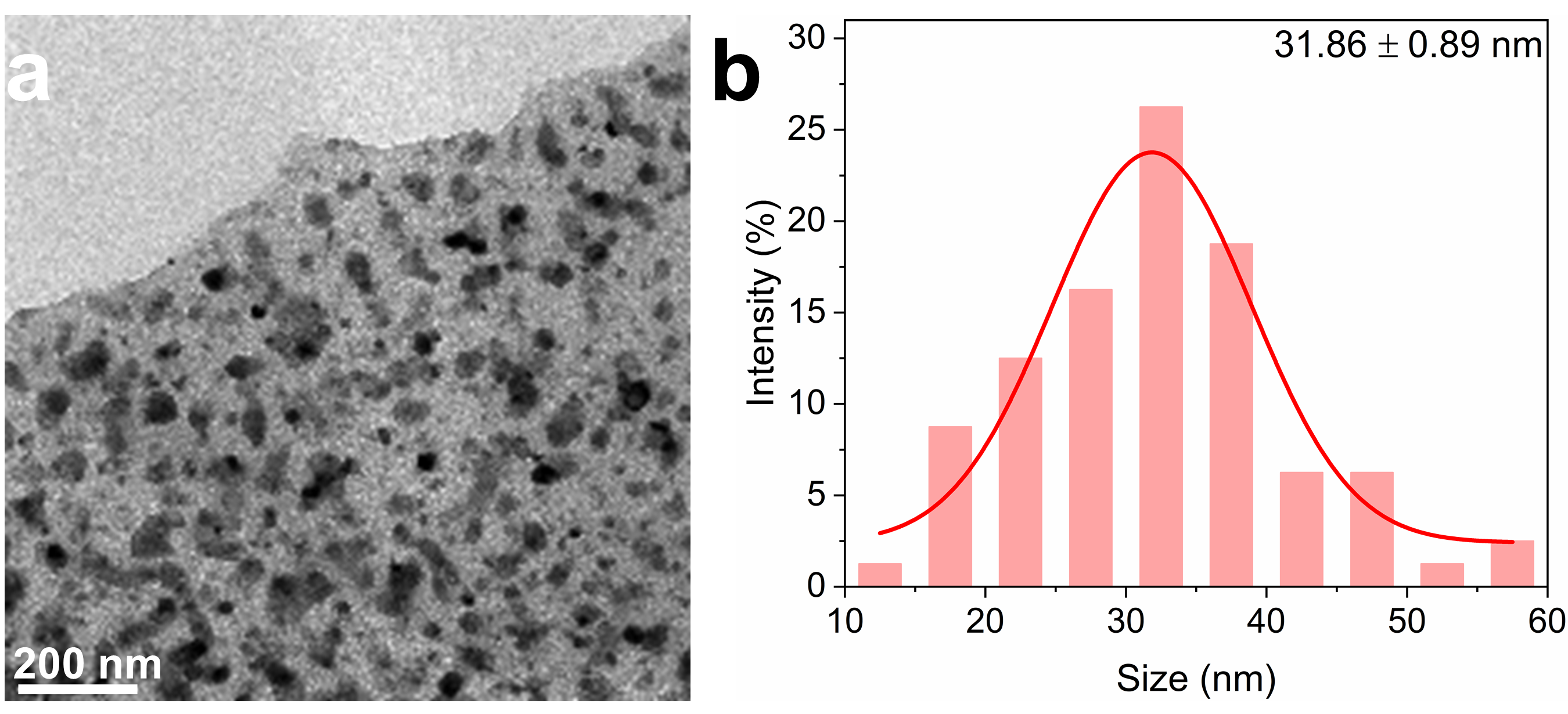


**Figure S7** TEM image of P-C/Z@C and corresponding size distribution.





**Figure S8** (a) SEM, (b) TEM, (c) HRTEM images, and (d) element mappings of C/Z@C.


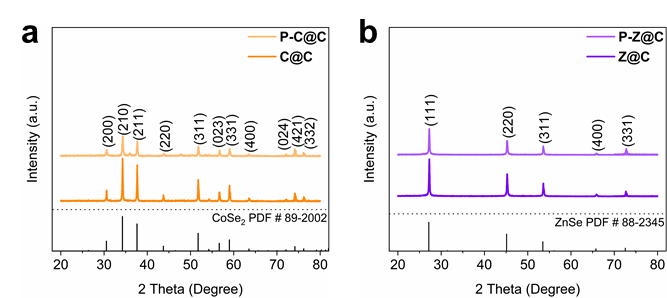


**Figure S9** XRD pattern of (a) P-C@C, C@C, and (b) P-Z@C, Z@C.





**Figure S10** (a) SEM image and (b-e) EDS mappings of P-C@C.





**Figure S11** (a) SEM image and (b-e) EDS mappings of P-Z@C.


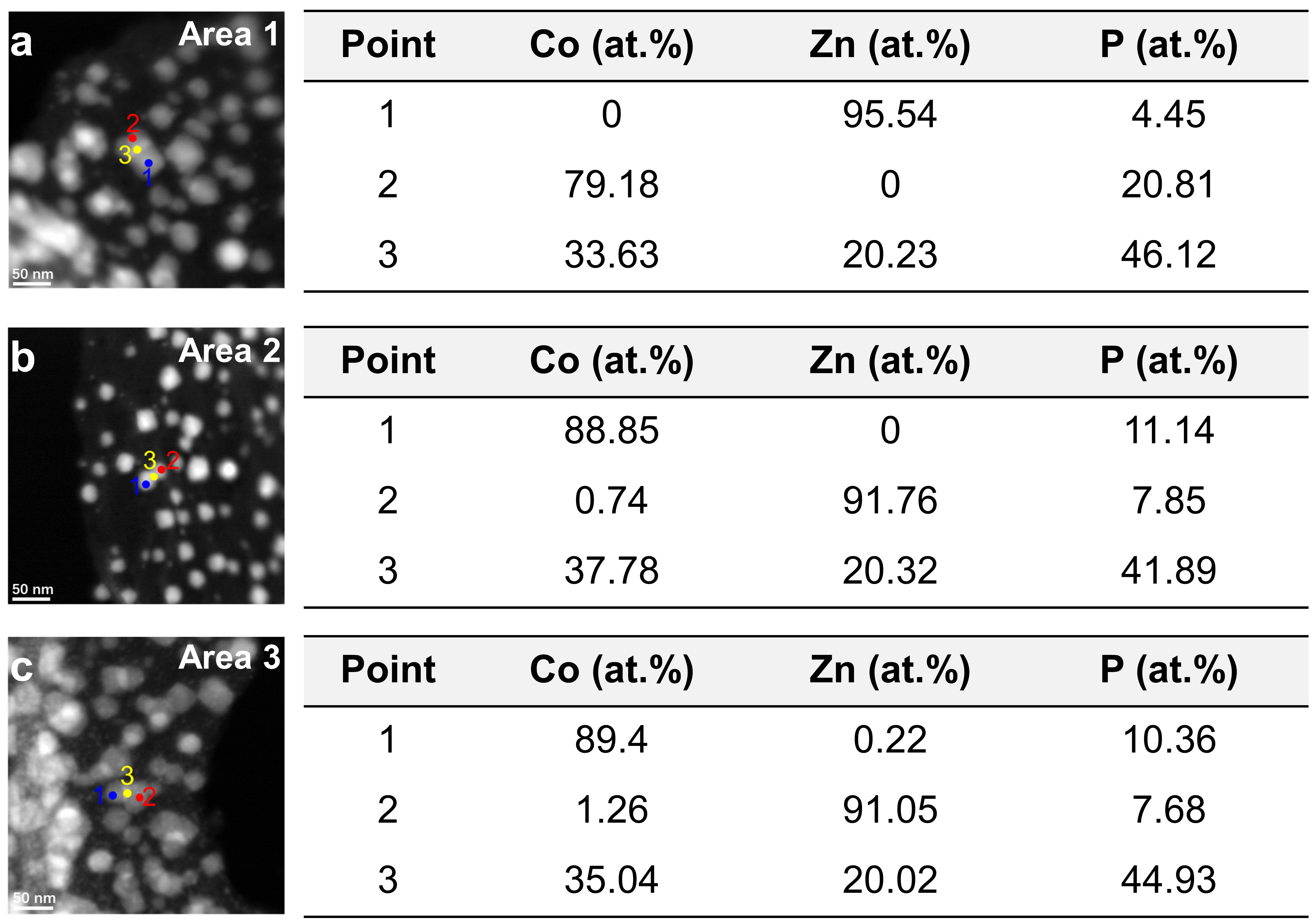


**Figure S12** TEM images of P-C/Z@C and corresponding element contents for Area 1, Area 2, Area 3, and Area 4 through point scan.





**Figure S13** rate performance of (a) P-C@C *vs*. C@C and (b) P-Z@C *vs*. Z@C.


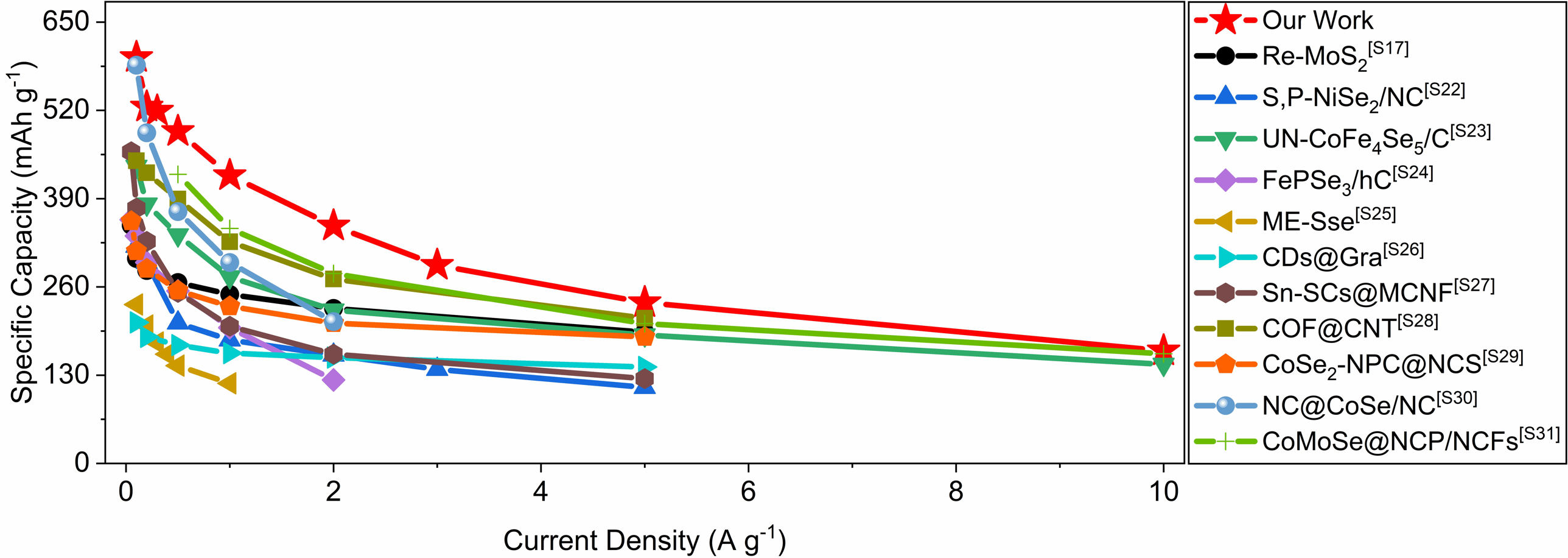


**Figure S14** Comparison of rate performance between P-C/Z@C and other literature reports.





**Figure S15** GCD curves of (a) P-C/Z@C and (b) C/Z@C at 0.1 A g^−1^.





**Figure S16** GCD curves of (a) P-C@C and (b) C@C at 0.1 A g^−1^.





**Figure S17** GCD curves of (a) P-Z@C and (b) Z@C at 0.1 A g^−1^.





**Figure S18** GITT curves of C/Z@C.





**Figure S19** Local enlarged GITT curves of P-C/Z@C





**Figure S20** CV curves of (a) C/Z@C and (b) P-C/Z@C at a scan rate of 0.1 mV s^–1^.





**Figure S21** Capacitive contribution ratio of (a) C/Z@C and (b) P-C/Z@C at a scan rate of 2 mV s^–1^.





**Figure S22** Ex-situ Co 2p XPS spectra of P-C/Z@C.





**Figure S23** IFFT image and corresponding lattice distance based on **Figure 5d**.





**Figure S24** IFFT image and corresponding lattice distance based on **Figure 5e**.





**Scheme S1** Schematical illustration of structure evolution for P-Z/C@C upon charge and discharge.


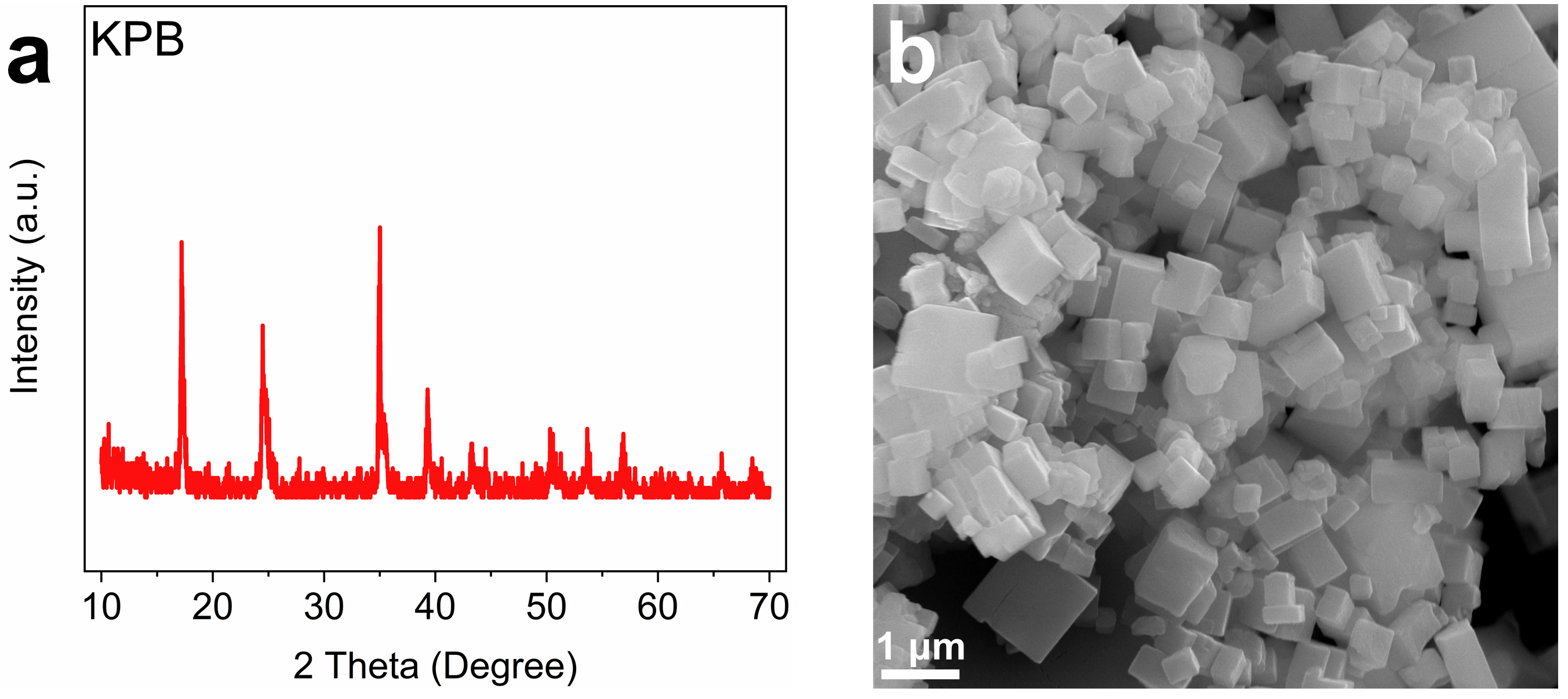


**Figure S25** (a) XRD pattern and (b) SEM image of KPB.

**Reference**

[S1] X. Zhang, F. Tian, X. Lan, Y. Liu, W. Yang, J. Zhang and Y. Yu, *Chem. Eng. J.*, **2022**, 429, 132588.

[S2] C. Peng, L. Song, L. Wang, F. Yang, J. Ding, F. Huang and Y. Wang, *ACS Appl. Energy Mater.*, **2021**, 4, 4887.

[S3] Y. Wei, X. Zhang, Z. Zhao, H.-S. Chen, K. Matras-Postolek, B. Wang and P. Yang, *Electrochim. Acta*, **2019**, 297, 553.

[S4] L. Bian, W. Gao, J. Sun, M. Han, F. Li, Z. Gao, L. Shu, N. Han, Z. x. Yang, A. Song, Y. Qu and J. C. Ho, *ChemCatChem*, **2018**, 10, 1571.

[S5] H. Lin, S. Zhang, T. Zhang, H. Ye, Q. Yao, G. W. Zheng and J. Y. Lee, *Adv. Energy Mater.*, **2019**, 9, 1902096.

[S6] F. Liu, N. Wang, C. Shi, J. Sha, L. Ma, E. Liu and N. Zhao, *Chem. Eng. J.*, **2022**, 431, 133923.

[S7] L. Zhang, J. Li, Z. Yang, L. Sun, G. Chen, W. An, H. Cheng, H. Wang, X. Wang, Y. Chen and F. Ma, *ChemistrySelect*, **2021**, 6, 1305.

[S8] Y. Gao, X. Yue, Y. Dong, Q. Zheng and D. Lin, *J. Colloid Interface Sci.*, **2024**, 658, 441.

[S9] R. A. Senthil, J. Pan, Y. Wang, S. Osman, T. R. Kumar and Y. Sun, *Ionics*, **2020**, 26, 6265.

[S10] Q. Wang, Z. Qu, S. Chen and D. Zhang, *J. Colloid Interface Sci.*, **2022**, 624, 385.

[S11] C. Ouyang, X. Wang and S. Wang, *Chem. Commun.*, **2015**, 51, 14160.

[S12] D. Nam, E. Jang and J. Kim, *J. Alloys Compd.*, **2023**, 947, 169625.

[S13] C. Wang, K. Xian, S. Zhao, S. Ma, H. Qin, D. Wang, A. Tayal, Y. Wang, Y.-W. Lin, M. Feng, X. Ou, *Nano Energy,* **2023**, 118, 109020.

[S14] S. Liang, Z. Yu, T. Ma, H. Shi, Q. Wu, L. Ci, Y. Tong, J. Wang, Z. Xu, *ACS Nano,* **2021**, 15, 14697.

[S15] S. Wu, X. Li, Y. Zhang, H. Fan, *Chem. Eng. Sci.,* **2025**, 313, 121737.

[S16] X. Zhang, S. Tian, S. Liu, T. Wang, J. Huang, P. Gao, Y. Feng, J. Zhou, T. Zhou, *Appl. Phys. Lett.,* **2024**, 125, 263904.

[S17] Y. Dong, S. Tian, P. Gao, F. Ma, S. Liu, T. Zhou, J. Zhou, *J. Energy Storage,* **2025**, 118, 116227.

[S18] H. Shan, J. Qin, J. Wang, H. M. K. Sari, L. Lei, W. Xiao, W. Li, C. Xie, H. Yang, Y. Luo, G. Zhang, X. Li, *Adv. Sci.,* **2022**, 9, 2200341.

[S19] H. N. Fan, X. Y. Wang, H. B. Yu, Q. F. Gu, S. L. Chen, Z. Liu, X. H. Chen, W. B. Luo, H. K. Liu, *Adv. Energy Mater.,* **2020**, 10, 1904162.

[S20] X. Luo, J. Huang, L. Cao, J. Li, Z. Xu, K. Kajiyoshi, Y. Zhao, H. Yang, Y. Liu, Z. Li, *Chem. Eng. J.,* **2023**, 464, 142579.

[S21] L. Song, S. Zhang, L. Duan, R. Li, Y. Xu, J. Liao, L. Sun, X. Zhou, Z. Guo, *Angew. Chem. Int. Ed.,* **2024**, 63, e202405648.

[S22] J. Ye, Z. Chen, Z. Zheng, Z. Fu, G. Gong, G. Xia, C. Hu, *J. Energy Chem.,* **2023**, 78, 401.

[S23] L. Song, S. Zhang, L. Duan, R. Li, Y. Xu, J. Liao, L. Sun, X. Zhou, Z. Guo, *Angew. Chem. Int. Ed.*, **2024**, 63, e202405648.

[S24] X.-H. Wu, B.-C. Chen, Z.-Q. Gu, X. Lu, H.-Y. Zhong, P.-W. Huang, J. Zhang, X.-Y. Tan, Y. Zhao, *Chem. Eng. J.,* **2025**, 506, 160266.

[S25] X.-H. Wu, L.-B. Yang, M.-J. Zhao, M.-R. Xu, W.-J. Jiang, B.-J. Feng, J.-J. Liu, Y. Zhao, *J. Mater. Chem. A*, 2025, 13, 11505.

[S26] Z. Qin, P. Xia, X. Liu, X. Jin, S. Lu, Y. Zhang, H. Fan, *Chem. Eng. J.*, **2025**, 121, 116628.

[S27] L. Li, A. Huang, H. Jiang, Y. Li, X. Pan, T.-Y. Chen, H.-Y. Chen, S. Peng, *Angew. Chem. Int. Ed.*, **2024**, 63, e202412077.

[S28] D. Yan, Dr. L. Song, F. Kang, X. Mo, Y. Lv, J. Sun, H. Tang, X. Zhou, Q. Zhang, *Angew. Chem. Int. Ed.*, **2025**, 64, e202422851.

[S29] Q. Liu, X. Tan, X. Li, Y. Li, X. Han, S. Cui, D. Xu, Y. Liu, R. Wang, Q. Zhao, M. Wu, *J. Energy Storage,* **2024**, 87, 111449.

[S30] D. Zhang, J. Xu, H. Sun, Z. Li, Q. Wang, Q. Sun, B. Wang, *Electrochim. Acta*, **2024**, 474, 143524.

[S31] Z. M. Yu, J. H. Jia, G. Y. Wang, Z. Wen, C. C. Yang, Q. Jiang, *ChemSusChem*, **2025**, 18, e202402434.
